# Dehydration before Major Urological Surgery and the Perioperative Pattern of Plasma Creatinine: A Prospective Cohort Series

**DOI:** 10.3390/jcm10245817

**Published:** 2021-12-13

**Authors:** Lukas M. Löffel, Dominique A. Engel, Christian M. Beilstein, Robert G. Hahn, Marc A. Furrer, Patrick Y. Wuethrich

**Affiliations:** 1 Department of Anaesthesiology and Pain Medicine, Inselspital, Bern University Hospital, University of Bern, CH 3010 Bern, Switzerland; lukas.loeffel@insel.ch (L.M.L.); dominique.engel@insel.ch (D.A.E.); christian.beilstein@insel.ch (C.M.B.); 2 Research Unit, Södertälje Hospital, Södertälje, Karolinska Institutet at Danderyds Hospital (KIDS), 18288 Stockholm, Sweden; robert.hahn54@gmail.com; 3 Department of Urology, Inselspital, Bern University Hospital, University of Bern, CH 3010 Bern, Switzerland; marcalain.furrer@outlook.com

**Keywords:** dehydration, gastrointestinal function, major urologic surgery, perioperative plasma creatinine pattern

## Abstract

Preoperative dehydration is usually found in 30–50% of surgical patients, but the incidence is unknown in the urologic population. We determined the prevalence of preoperative dehydration in major elective urological surgery and studied its association with postoperative outcome, with special attention to plasma creatinine changes. We recruited 187 patients scheduled for major abdominal urological surgery to participate in a single-center study that used the fluid retention index (FRI), which is a composite index of four urinary biomarkers that correlate with renal water conservation, to assess the presence of dehydration. Secondary outcomes were postoperative nausea and vomiting (PONV), return of gastrointestinal function, in-hospital complications, quality of recovery, and plasma creatinine. The proportion of dehydrated patients at surgery was 20.4%. Dehydration did not correlate with quality of recovery, PONV, or other complications, but dehydrated patients showed later defecation (*p* = 0.02) and significant elevations of plasma creatinine after surgery. The elevations were also greater when plasma creatinine had increased rather than decreased during the 24 h prior to surgery (*p* < 0.001). Overall, the increase in plasma creatinine at 6 h after surgery correlated well with elevations on postoperative days one and two. In conclusion, we found preoperative dehydration in one-fifth of the patients. Dehydration was associated with delayed defecation and elevated postoperative plasma creatinine. The preoperative plasma creatinine pattern could independently forecast more pronounced increases during the early postoperative period.

## 1. Introduction

The degree of preoperative dehydration and its relationship to postoperative outcome is unclear. A particular reported difficulty when studying these issues is obtaining adequate power for statistical significance [1]. The optimal choice of diagnostic remains a matter of debate. A raised serum osmolality is most commonly used to detect a deficit of body water, while concentrated urine appears to be a simpler and even more sensitive alternative [2,3,4]. The reported incidence of preoperative dehydration, when based on urine sampling, is approximately one-third to half of all patients in different surgical populations [5,6,7]. The prevalence in the urologic population is not presently known, but it is relevant because an adequate fluid regime in the perioperative period might reduce complications [8,9]. Some studies show that preoperative dehydration increases the risk of overall postoperative complications, while others indicate a negative effect on morbidity, for example, by causing postoperative nausea and vomiting (PONV) [1,5]. Other studies have been unable to show these effects [10].

The objective of this observational study was to determine the prevalence of preoperative dehydration, as measured by the fluid retention index (FRI), among candidates for elective major urologic surgery and its relationship to postoperative outcome. FRI is a summary measure of several indices of concentrated urine that aims to provide a robust assessment of renal water conservation [10]. We collected data on the incidence of surgery-associated nausea and vomiting, postoperative return of gastrointestinal function, fluid balance, complications within hospitalization, quality of recovery, and renal function. Special emphasis was placed on the perioperative pattern of plasma creatinine.

## 2. Materials and Methods

### 2.1. Approvals

This monocentric observational study reports a single tertiary center cohort series, and accords with the Strengthening the Reporting of Observational Studies in Epidemiology (STROBE) recommendations. Ethical approval was provided by the Ethics Committee of the Canton Bern, Switzerland (KEK Bern, Project-ID 2018-01804, Chairperson Professor C. Seiler) on 3 December 2018, and the study was registered at ClinicalTrials.gov (accessed on 21 December 2018) with the identifier NCT03788070. Written informed consent was obtained from all participants.

### 2.2. Study Population and Design

The study goal was to consecutively identify and include patients at the Department of Urology of the University Hospital Bern undergoing major abdominal urologic surgery. Inclusion started on 11 February 2019 and ended on 16 March 2020 (due to the COVID-19 crisis), and comprised patients scheduled for elective major urologic laparotomy or robotic assisted laparoscopy, which included prostatectomy, bladder and kidney surgery, reconstruction of the ureter, and open retroperitoneal lymph node dissection. Exclusion criteria were preoperative need for dialysis, preoperative IV fluid administration, age < 18 years, pregnancy, and enrollment of employees and other dependent persons.

### 2.3. Outcome Measures

The primary outcome was the prevalence of dehydration before the surgery, as defined by the fluid retention index (FRI), which is the mean score of four urine measurements that reflect renal water conservation, considered consistent with dehydration. A patient is deemed dehydrated if the FRI is ≥4.0; this cut-off corresponds to a urine-specific gravity of ≥1.020, urine osmolality ≥600 mosmol/kg, urine creatinine ≥12 mmol/L, and urine color ≥4, as assessed visually on a standardized color chart (Table 1) [4,7,11].

Secondary outcomes were PONV, return of gastrointestinal function, intra- and postoperative fluid balance, complications during hospitalization (Appendix A), quality of recovery judged by the quality of recovery-15 (QoR-15) questionnaire, and postoperative renal function, as indicated by repeated measurements of plasma creatinine [12].

### 2.4. Data Collection and Laboratory Tests

Urine and plasma samples were collected the day before surgery (at around 2 p.m.; time point defined as “preoperative”), at the start of surgery (directly after induction of anesthesia, time point defined as “surgery”), 6 h after surgery ended, and at 6 a.m. on postoperative day one (POD1) and postoperative day two (POD2). Urine samples were taken directly from the ureteral catheters if present (bladder and kidney surgery) or from the urethral catheter.

Urine samples were analyzed for specific gravity, osmolality, creatinine, and color. The plasma was analyzed for osmolality, creatinine, sodium, potassium, hemoglobin, and hematocrit. The measurements were performed by the certified clinical chemistry laboratory at our hospital (osmolality: Station 6060 TT, Array Global Business Inc., Japan, and urine/plasma electrolytes: COBAS 8000/ISE Modul, Roche Diagnostics, Basel, Switzerland). Urine specific gravity was determined with a digital photometric refractometer, which expresses values in steps of 0.005 based on the reflection coefficient of light directed onto strips dipped in urine (Uricon-Ne, Atago CO. LTD., Tokyo, Japan).

A dedicated study nurse assessed PONV, based on a verbal rating scale and emetic episodes, at the same time as laboratory samples were drawn. Data on onset of flatus, defecation, and the quality of recovery (QoR 15 questionnaire) were also obtained. Intraoperative fluid administration and blood loss, fluid balance (difference in body weight between preoperative and POD1), and complications during hospitalization (Bennett Guerrero score) were assessed from the hospital medical records [13].

### 2.5. Perioperative Management

Pre-operative oral hydration including carbohydrate-loading beverages was allowed up to 2 h before the start of anesthesia. No enteral bowel preparation and no enemas were performed preoperatively. Patients were allowed to drink clear fluids immediately after surgery while in the intermediate care unit. An oral liquid diet was started on POD1. Perioperative patient management was based on an Enhanced Recovery Approach [14,15]. PONV prophylaxis was based on preoperative Apfel score assessment, and performed in accordance with the consensus recommendation [16,17].

Surgery was performed under general anesthesia. After induction with propofol (1–2 mg/kg), rocuronium (0.6–0.9 mg/kg), and fentanyl (2 µg/kg), patients were ventilated with tidal volumes of 6 mL/kg ideal body weight and a positive end-expiratory pressure (PEEP) of 5–10 mmHg, depending on body mass index (BMI). Baseline plasma volume support consisted of fluid administration of Ringer’s lactate at a rate up to 4 mL/kg/h. If hypotension was observed (mean arterial pressure <60 mmHg), norepinephrine was titrated to a maximum of 8 μg/kg/h after an initial bolus of 5–10 μg. A fluid bolus of 250 mL of lactated Ringer’s was administered in cases where the patient was judged to be hypovolemic, based on pulse pressure variation > 10% [8,18].

Blood loss was obtained as the sum of the volume present in the suction bag and the weight of sponges and swabs. Hemorrhage up to 500 mL was replaced with an equal amount of Ringer’s lactate. Packed red blood cells were transfused if blood hemoglobin fell below 80 g/L (<100 g/L in patients with severe coronary artery disease). No hydroxyethyl starch solution was given.

### 2.6. Statistical Analysis

Continuous variables were presented as mean ± standard deviation if normally distributed. Variables with a skewed distribution were reported as the median (25–75% interquartile range, IQR). Categories were presented as numbers and percentages.

A binomial test and confidence interval were performed to assess the primary outcome at surgery. Secondary outcomes were assessed longitudinally with a Brunner and Langer non-parametric repeated-measures ANOVA, followed by Mann–Whitney tests post hoc [19]. Group-wise comparisons of secondary outcomes were assessed using Mann–Whitney and Fisher’s exact tests. *p* < 0.05 was considered statistically significant. Relationships between creatinine values at different time points were evaluated by correlation analysis. A two-way ANOVA, based on (1 + log) transformed values due to skewed distribution, was performed for point-wise analysis of dehydration and the preoperative change in plasma creatinine.

All *p*-values for post hoc tests of secondary outcomes and in the retrospective analysis of creatinine ratios were corrected by the Holm method. All analyses in this report were performed with the statistics software R, version 3.5.0.

No formal power analysis was performed, as the incidence and severity of dehydration in patients undergoing major urological surgery was still unknown when the study was initiated. However, based on other types of surgery, we expected to recruit approximately 200 patients (with a variance of ±10%) [5].

## 3. Results

We screened 238 consecutive patients scheduled for elective major urological surgery. We initially recruited 193 patients, but five dropped out before the study began (three patients due to cancellation of the surgery, one patient for logistical reasons, and one patient for preoperative dialysis). One patient withdrew consent after inclusion, leaving 187 to provide data. Overall, six patients (3.2%) had missing values for the assessment of our primary outcome due to logistical reasons, resulting in 181 patients included in the final analysis of dehydration as the primary endpoint (Supplementary Materials Figure S1).

### 3.1. Dehydration

The proportion of dehydrated patients at surgery was 20.4% (37/181 patients, 95% CI: 14.8–27.1%). A larger fraction of the prostate surgery patients were dehydrated (40.5%; *p* = 0.005), but other differences in demographics, preoperative health status, and chronic medication were only occasional and slight between hydrated and dehydrated patients (Table 2). The two groups received similar amounts of fluid during the surgeries, with an overall volume of administered crystalloid of 1.3 (1.1–2.1) L, infused at a rate of 4.5 (3.0–6.5) mL/kg/h.

The outcome data showed no statistically significant differences between the groups for blood loss, fluid balance on POD1, in-hospital complications, and the quality of recovery (QoR-15 questionnaire) (Table 3). No differences were noted in the occurrence of flatus (*p* = 0.48) and defecation (*p* = 0.61); however, defecation occurred later in the dehydrated patients (*p* = 0.02). Twice as many dehydrated patients had PONV on POD1 (22% vs. 11%), but the difference over time was not significant (*p* = 0.48; Table 4).

The perioperative increase in plasma creatinine was also greater in the dehydrated than in the hydrated patients (Table 5, top).

### 3.2. Trajectory of Plasma Creatinine

The preoperative plasma creatinine concentration was 81 (68–90) µmol/L. The time course showed that the final postoperative nadir value could be forecasted from the measurement performed at 6 h after surgery, but patients experiencing a rise in plasma creatinine between the preoperative measurement and the sample taken just before surgery started were more likely to have a greater postoperative elevation of plasma creatinine (Table 5, lower section).

**Figure 1:**
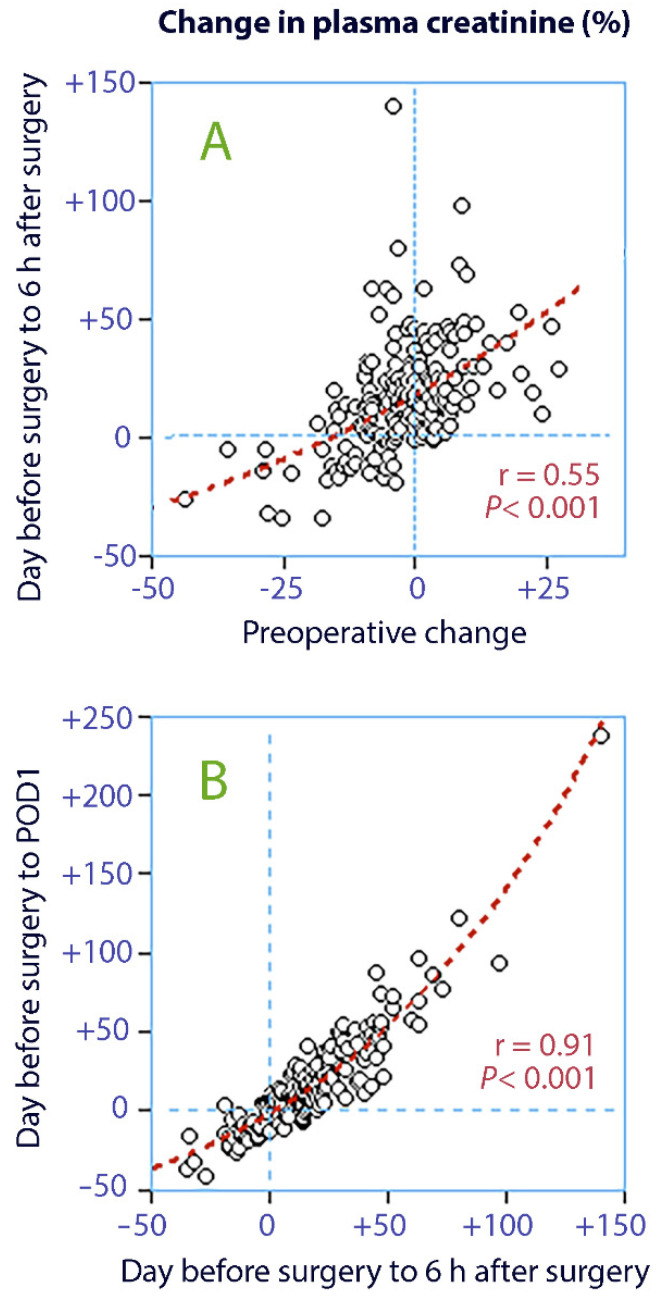
Linear correlations between changes in plasma creatinine; (**A**) the preoperative change vs. the change from the day before surgery to 6 h after surgery; (**B**) the further change to the first postoperative day (POD1).

The correlations suggest that 30% of the variability in plasma creatinine at 6 h after surgery could be accounted for by the preoperative change (Figure 1A), and that 83% of the variability on POD1 could be explained by the change that had occurred by 6 h (Figure 1B).

**Figure 2:**
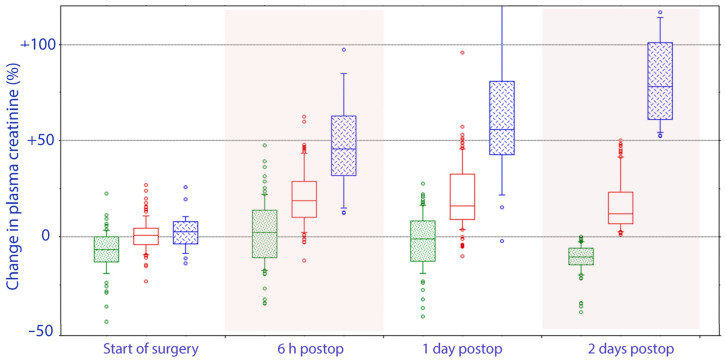
Perioperative trajectory of plasma creatinine based on whether the final postoperative value represented a decrease in plasma creatinine values (green), a moderate increase (0–50%; red) or a substantial increase (>50%, blue) as compared to the concentration measured on the day before surgery. A statistically significant difference between these groups had occurred already before surgery was initiated, i.e., from the day before the surgery to the onset of surgery.

Approximately two-thirds of the patients had a reduction in plasma creatinine between POD1 and POD2. The patients with the greatest increase on POD1 showed a further increase, while those with a less pronounced increase had a decrease (difference, *p* < 0.003; Figure 2).

### 3.3. Risk Factors for Plasma Creatinine Elevation

Two-way analysis of variance showed that both dehydration and the preoperative change in plasma creatinine served as statistically significant and independent predictors of the elevation of plasma creatinine at 6 h (*p* < 0.05 and *p* < 0.001, respectively) and on POD1 (*p* < 0.012 and *p* < 0.001), while only the preoperative change in plasma creatinine was significant on POD2 (*p* = 0.006).

**Figure 3:**
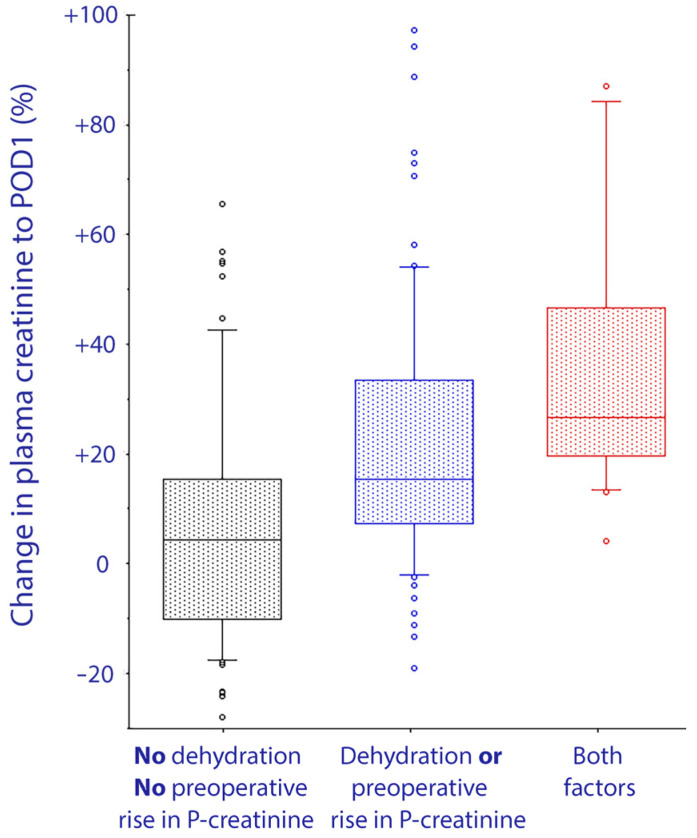
Change in plasma creatinine (as a percentage) from the day before surgery to the first day after surgery, depending on whether patients were dehydrated and/or showed an increase in plasma creatinine from the day before surgery to just before the surgery started. The box shows the 25th, 50th, and 75th percentiles and the error bars indicate the 10th and 90th percentiles.

The median rise in plasma creatinine never exceeded 5% for the group of 82 patients who were both well hydrated and showed a preoperative reduction in plasma creatinine. By contrast, the 18 patients who were both dehydrated and had a preoperative rise in plasma creatinine showed a median increase of 26% on POD1, and seven had elevations of 40% or more (Figure 3).

Exploratory analyses suggested that a body mass index >30 kg/m^2^ (*n* = 43) served as an additional independent predictor that would strengthen the presented correlations. However, we considered that the subgroups would then be too small for presentation.

## 4. Discussion

### 4.1. Key Results

The proportion of dehydrated patients was 20.4%, which was lower than expected, as earlier studies reported a prevalence of 30–50% [5,6,7]. Preoperative dehydration was not significantly associated with PONV, fluid balance, or quality of recovery score (QoR15), but dehydrated patients were prone to delayed defecation and postoperative elevations of plasma creatinine. Interestingly, our study of the trajectory showed that the changes in plasma creatinine that occurred while awaiting surgery were also associated with later changes. To our knowledge, this observation has not been reported previously. Preoperative dehydration and this early change in plasma creatinine both appeared to serve as independent risk factors for postoperative elevations of plasma creatinine. Their strength can be illustrated by the fact that one-third of those who presented with dehydration and a 24 h rise in plasma creatinine had a later postoperative increase of 40% or more. By contrast, elevations of 5–10% were rare in patients who did not present with these characteristics.

### 4.2. Interpretation

The later defecation in the dehydrated patients is probably explained by more enteral water resorption and a consequently reduced amount of feces of harder consistency. This agrees with previous findings of a general statistical link between dehydration and obstipation [20].

The dehydration diagnosis we used implies that the kidneys were set to conserve water when surgery began. The additional renal fluid retention induced by anesthesia and surgery could then be troublesome for creatinine excretion. The maximum renal capacity for creatinine excretion is not precisely known, but is likely to vary among individuals.

We hypothesize that fluid retention caused by preoperative stress surpassed the renal threshold for creatinine excretion in those who showed a rise in plasma creatinine before the surgery [21]. These patients probably had a reduced capacity to excrete creatinine due to pre-existing limitations of kidney function. By contrast, dehydration had a less pronounced effect on postoperative plasma creatinine in patients who did not show this rise before the surgery.

### 4.3. Literature

Several forms of dehydration are recognized, but the patients diagnosed as being dehydrated in the present study had most certainly ingested very little water just before surgery or had a habitually low intake of water (intracellular dehydration) [2,21]. Chronic dehydration is a challenging diagnosis, and a universally accepted standard reference biomarker is still lacking. Serum osmolality is often recommended; however, hyperosmolality seems to require that the kidneys fail to concentrate the urine [22]. We used urine analysis for the present study, as it has been well evaluated in sports medicine and occasionally applied in hospital care [3,7].

Concentrated urine is common in the general population. Only a few studies have suggested that (chronic) dehydration has an impact on health outcomes [11,23]. Concentrated urine is associated with a high 30-day mortality in acute geriatric care and a higher rate of complications after hip fracture surgery [5,24]. Concentrated urine changes the body´s handling of both crystalloids and colloids in the sense that more fluid is needed to perform fluid optimization before surgery [6,25,26]. In addition, treatment of dehydration if enteral fluid administration is not sufficient or possible (i.e., early postoperative period), the administration of hypotonic solution (glucose 5% for example) is of importance. Salt load has been also presented as a risk factor for fluid accumulation during the early postoperative phase [27].

Postoperative acute kidney injury (AKI) is a postoperative complication most commonly diagnosed based on a perioperative rise in plasma creatinine. The current criteria (kidney disease: Improving Global Outcomes: KDIGO) for Stage 1 AKI are fulfilled if the plasma creatinine increases by 50%, compared to the baseline [28]. The medical community has shown great concern regarding this complication, since chronic kidney injury occasionally ensues and increases morbidity and mortality [29,30].

### 4.4. Future Views

The mechanisms we suggest do not rest on the occurrence of perioperative harm to the kidneys, but on normal physiological responses to low fluid intake, perioperative stress, and minor pre-existing limitations of renal function. Nevertheless, our findings could raise the possibility of perioperative management in high-risk patients that would predict the risk and possibly prevent substantial postoperative increases in plasma creatinine, and perhaps even acute kidney injury, through urine analysis and preoperative measurement of plasma creatinine.

### 4.5. Strengths and Limitations

One strength of this study is that we included a large sequential cohort of major abdominal urological surgery patients over a short time period. All patients were managed pre-, intra-, and post-operatively in a nearly identical manner, as the perioperative pathway is standardized according to the type of surgery in our institution [8,18]. A limitation of this study was its prospective assessment of data from a single high caseload center, as this could limit the possibility of generalizing the findings. Another possible limitation is that we did not assess neutrophil gelatinase-associated lipocalin (NGAL) perioperatively, and the postoperative increase in plasma creatinine has been shown to be preceded by an elevation of NGAL. However, NGAL elevation itself was linked to the high fluid retention index, suggesting dehydration or mechanisms inducing fluid retention [7].

An additional limitation is the lack of precise hemodynamic information, since we are not able to exclude variations in cardiac output (i.e., low flow situations) during the observation period, despite adequate (invasive arterial blood pressure) monitoring and standard procedures to combat hypovolemia. However, the expectation is that all patients would have been treated the same way (with additional fluid administration and vasopressors) to combat hypotension and hypovolemia, as both are known risk factors for acute kidney injury [31].

## 5. Conclusions

Preoperative dehydration was present in one-fifth of the patients. Dehydration was not associated with postoperative complications, PONV or differences in quality of recovery assessed by the QoR15. However, dehydrated patients were prone to delayed defecation and a postoperative rise in plasma creatinine. Even greater elevations of plasma creatinine occurred in patients who also showed a rise in plasma creatinine during the 24 h prior to the operation.

## Figures and Tables

**Table 1:** Description of the four dimensions of the fluid retention index (FRI).

Score	1	2	3	4	5	6
Specific gravity	≤1.005	1.010	1.015	1.020	1.025	1.030
Osmolality (mOsmol/kg)	<250	250–450	450–600	600–800	800–1000	>1000
Creatinine (mmol/L)	<4	4–7	7–12	12–17	17–25	>25
Color (shade)	1	2	3	4	5	6

**Table 2:** Patient’s pre-, intra-, and postoperative data. Dehydration grouping according to fluid retention index (FRI) ≥ 4 obtained when surgery was initiated. Data shown as median (25th–75th percentiles) or block column percentages depending on distribution. ^+^ values measured at surgery. Missing data coming from patients in the hydrated group.

	All Patients *n* = 181	Dehydrated *n* = 37	Hydrated *n* = 144	*p*-Value
Type of Surgery				
Prostate *n* (%)	44 (24.3%)	15 (40.5%)	29 (20.1%)	0.005
Bladder *n* (%)	50 (27.6%)	3 (8.1%)	47 (32.6%)	
Kidney *n* (%)	51 (28.2%)	11 (29.7%)	40 (27.8%)	
Other *n* (%)	36 (19.9%)	8 (21.6%)	28 (19.4%)	
Open/minimal invasive *n* (%)	130 (72%)/51 (28%)	23 (62%)/14 (38%)	107 (74%)/ 37 (26%)	0.22
Age (years)	65.0 (56.5–71.0)	62.0 (51.0–68.0)	65.5 (56.8–72.0)	0.04
BMI (kg/height^2^)	26.9 (23.9–29.7)	29.0 (24.2–32.5)	26.6 (23.9–29.1)	0.04
ASA 1 *n* (%)	9 (5%)	3 (8.1%)	6 (4.2%)	0.16
ASA 2 *n* (%)	81 (44.8%)	21 (56.8%)	60 (41.7%)	
ASA 3 *n* (%)	89 (49.2%)	13 (35.1%)	76 (52.8%)	
ASA 4 *n* (%)	2 (1.1%)	0 (0.0%)	2 (1.4%)	
Ischemic heart disease *n* (%)	26 (14.5%)	3 (8.3%)	23 (16.1%) **	0.30
Hypertension *n* (%)	86 (47.5%)	19 (51.4%)	67 (46.5%)	0.71
Diabetes mellitus *n* (%)	18 (9.9%)	5 (13.5%)	13 (9.0%)	0.54
Smoking *n* (%)	54 (31%)	17 (45.9%)	37 (25.6%)	0.03
GFR > 90 mL/min	71 (39.7%)	21 (56.8%)	50 (35.2%) **	<0.0001
GFR 60–89 mL/min	63 (35.2%)	16 (43.2%)	47 (33.1%)	
GFR < 60 mL/min	45 (25.1%)	0 (0.0%)	45 (31.7%)	
Betablocker *n* (%)	29 (16.2%)	7 (18.9%)	22 (15.5%) **	0.62
Calcium antagonists *n* (%)	79 (43.6%)	16 43.2%)	63 (43.8%)	1.00
Statins *n* (%)	44 (24.3%)	12 (32.4%)	32 (22.2%)	0.20
Aspirin *n* (%)	28 (15.6%)	8 (21.6%)	20 (14.0%)	0.31
Diuretics *n* (%)	17 (9.4%)	2 (5.4%)	15 (10.5%) **	0.53
Antidiabetics *n* (%)	17 (9.6%)	5 (13.5%)	12 (8.5%) **	0.35
Hemoglobin ^+^ (mmol/L)	135 (122–144)	140 (131–144)	133 (118–143) **	0.01
Osmolality ^+^ (mosmol/kg)	288 (284–291)	288 (285–290)	288 (283–291) **	0.65
Creatinine ^+^ (µmol/L)	84 (70–96)	73 (65–85)	85.5 (72–102) *	0.004
Intraoperative Ringer lactated (mL)	1300 (1050–2100)	1800 (1100–2100)	1300 (1025–2100)	0.35
Intraoperative Colloids (mL)	0 (0–0)	0 (0–0)	0 (0–0)	0.29
Intraoperative packed red blood cells (mL)	0 (0–0)	0 (0–0)	0 (0–0)	0.34
Intraoperative fresh frozen plasma (mL)	0 (0–0)	0 (0–0)	0 (0–0)	0.21
Blood Loss (mL)	400 (200–700)	400 (200–600)	400 (200–750)	0.90
Fluid balance on -POD1 (kg)	0.8 (−2.2–0.1)	1.0 (−2.7–0.0)	0.7 (−2.0–0.2)	0.35

*p*-values calculated using exact Fisher or exact Mann–Whitney Tests, depending on data distribution. Number of missing data: ** *n* = 2; * *n* = 1.

**Table 3:** Non-longitudinal secondary outcomes: Dehydration according to the fluid retention index (FRI) ≥ 4. Data are presented median (25th–75th percentiles) for numeric outcomes and percentages (95% CI) for categorical/dichotomous outcomes.

	All Patients	Dehydrated	Hydrated	*p*-Value
	*n* = 181	*n* = 37	*n* = 144	
Difference in QoR15 Scores				
A-Part (questions 1–10)	−25 (−34.0, −15.0)	−26 (−33.5, −16.0)	24.5 (−35.8, −14.0)	0.68
B-Part (questions 11–15)	−1.0 (−6.0, 2.0)	−2.0 (−5.0, 0.5)	−1.0 (−6.8, 2.8)	0.76
Overall (questions 1–15)	−27.0 (−38.0, −14.0)	−28.0 (−36.0, −17.0)	−25.5 (−38.8, −13.0)	0.55
In-hospital complications				
Bennett-Guerrero Score	1.0 (0.0, 1.0)	1.0 (0.0, 1.0)	1.0 (0.0, 1.0)	0.18
Cardiovascular Complications (%)	5.0 (2.3, 9.2)	2.7 (0.1, 14.2)	5.6 (2.4, 10.7)	0.69
Pulmonary Complications (%)	2.8 (0.9, 6.3)	2.7 (0.1, 14.2)	2.8 (0.8, 7.0)	1.00
Infections (%)	11.6 (7.3, 17.2)	5.4 (0.7, 18.2)	13.2 (8.1, 19.8)	0.26
Neurological Complications (%)	3.9 (1.6, 7.8)	0.0 (0.0, 9.5)	4.9 (2.0, 9.8)	0.35
Renal Complications (%)	20.4 (14.8, 27.1)	18.9 (8.0, 35.2)	20.8 (14.5, 28.4)	1.00
Gastrointestinal Complications (%)	9.4 (5.6, 14.6)	2.7 (0.1, 14.2)	11.1 (6.5, 17.4)	0.20
Postoperative Transfusion (%)	7.7 (4.3, 12.6)	0.0 (0.0, 9.5)	9.7 (5.4, 15.8)	0.08
Deaths (%)	0.6 (0.0, 3.0)	0.0 (0.0, 9.5)	0.7 (0.0, 3.8)	1.00

*p*-values were calculated using exact Fisher or exact Mann–Whitney tests, depending on data distribution.

**Table 4:** Longitudinal secondary outcomes at 6 h postoperatively on POD 1 and 2.

Variable	Group	6h postop.	POD 1	POD 2
PONV (%)	Dehydrated	16.2 (6.2, 32)	21.6 (9.8, 38.2)	8.1 (1.7, 21.9)
	Hydrated	15.3 (9.8, 22.2)	11.1 (6.5, 17.4)	10.5 (5.9, 16.6)
Flatus (hours)	Dehydrated	–	43.2 (27.1, 60.5)	91.9 (78.1, 98.3)
	Hydrated	–	47.6 (38.9, 55.7)	79.4 (70.1, 84.3)
Defecation * (%)	Dehydrated	–	2.7 (0.1, 14.2)	56.8 (39.5, 72.9)
	Hydrated	–	11.1 (6.5, 17.4)	43.0 (34.2, 50.9)
		–	*p* = 0.29	*p* = 0.29

* significant group factor or interaction in ANOVA including post hoc tests. Data are median (25th–75th percentiles) for numeric outcomes and percentages (95%-CI) for categorical/dichotomous outcomes.

**Table 5:** Statistical comparison between postoperative plasma creatinine relative to the preoperative concentration, depending on the presence of dehydration (as indicated by a preoperative urine sample) and whether plasma creatinine increased or decreased during the preoperative period.

Plasma Creatinine (% Change)	Dehydrated (*n* = 37)	Hydrated (*n* = 144)	*p*-Value
6 h/preop	20 (13–41)	13 (1–25)	0.01
POD 1/preop	23 (9–39)	9 (−3–25)	0.02
POD 2/preop	12 (−2–42)	3 (−9–18)	0.013
	**Preoperative change in P-creatinine (% change)**		
	≥1.0 (*n* = 82)	<1.0 (*n* = 102)	
6 h/preop	21 (13–36)	8 (−6–20)	0.001
POD 1/preop	18 (9–39)	6 (−8–21)	0.001
POD 2/preop	11 (1–27)	−2 (−12–12)	0.001

Data are the median and interquartile range. The Mann–Whitney U test was used for statistics.

## Data Availability

For confidentiality reasons, we are not able to make all data publicly available.

## Supplementary Materials

The following are available online at https://www.mdpi.com/article/10.3390/jcm10245817/s1, Figure S1: Flow chart with inclusion and exclusion criteria used in the analysis.

## Appendix A. Definitions of Postoperative Complications/Events

### Appendix A.1. Gastrointestinal Complications

- Ileus: no evidence of bowel function (no flatus and no passage of stool) with abdominal distension requiring cessation of oral intake and intravenous fluid support by POD 5;
- Constipation: no passage of stool without signs of ileus by POD 5;
- Gastric ulcer: diagnosis made by gastroscopy;
- Anastomotic bowel leak: considered as a complication if requiring surgery or prolonged drainage.

### Appendix A.2. Complications of Infections

- Urinary tract infection: temperature >38 °C in the last 24 h and leukocytosis and a prompted urinary analysis that showed bacterial counts >100,000 requiring antibiotics;
- Sepsis: bacterial infection and at least two of the following clinical signs: hypo- or hyperthermia, tachycardia, tachypnea, leukocytopenia or leukocytosis, positive blood culture;
- Pneumonia: temperature >38 °C and leukocytosis and clinical signs of pneumonia, requiring antibiotics;
- Wound infection: pus can be expressed or aspirated, requiring surgical intervention.

### Appendix A.3. Wound Complications

- Wound dehiscence: diagnosed clinically and requiring resuturing.

### Appendix A.4. Cardiac Events

- Myocardial infarction: increase of the enzyme high-sensitive troponin T above the hospital laboratory’s myocardial infarction threshold (>0.05 µg/L), and either new Q wave changes, or persistent changes in ST-T segments;
- Arrhythmia: confirmed by 12-lead electrocardiography and requiring new medication or electroconversion;
- Congestive heart failure and pulmonary edema: shortness of breath, rales, jugular venous distension, peripheral edema, third heart sound, radiologic signs (cardiomegaly, interstitial or alveolar edema), brain natriuretic peptide value >500 pg/mL and diagnosis requiring diuretics;
- Transient brain natriuretic peptide increase: postoperative serum brain natriuretic peptide values between 100 and 500 pg/mL (considered as minor cardiac event).

### Appendix A.5. Thromboembolic Complications

- Pulmonary embolism: evidenced by spiral computerized tomography scanning.

### Appendix A.6. Genitourinary Complications

- Renal dysfunction: transient increase of creatinine: creatinine >50% upper limit of normal value;
- Renal failure: severe reduction in glomerular filtration rate (15–29 mL·min^−1^ × 1.73 m^−2^) at discharge;
- Urinary leakage: radiologically diagnosed, requiring stenting.

### Appendix A.7. Neurological Complications

- Presence of a new focal deficit, confusion/delirium.
