# Supplementary material for: Dehydration before Major Urological Surgery and the Perioperative Pattern of Plasma Creatinine: A Prospective Cohort Series
Source: J Clin Med. 2021 Dec 13;10(24):5817. doi: 10.3390/jcm10245817 (PMC8706637; doi:10.3390/jcm10245817)

Supplementary Materials

Figure S1. Flow chart with inclusion and exclusion criteria used in the analysis

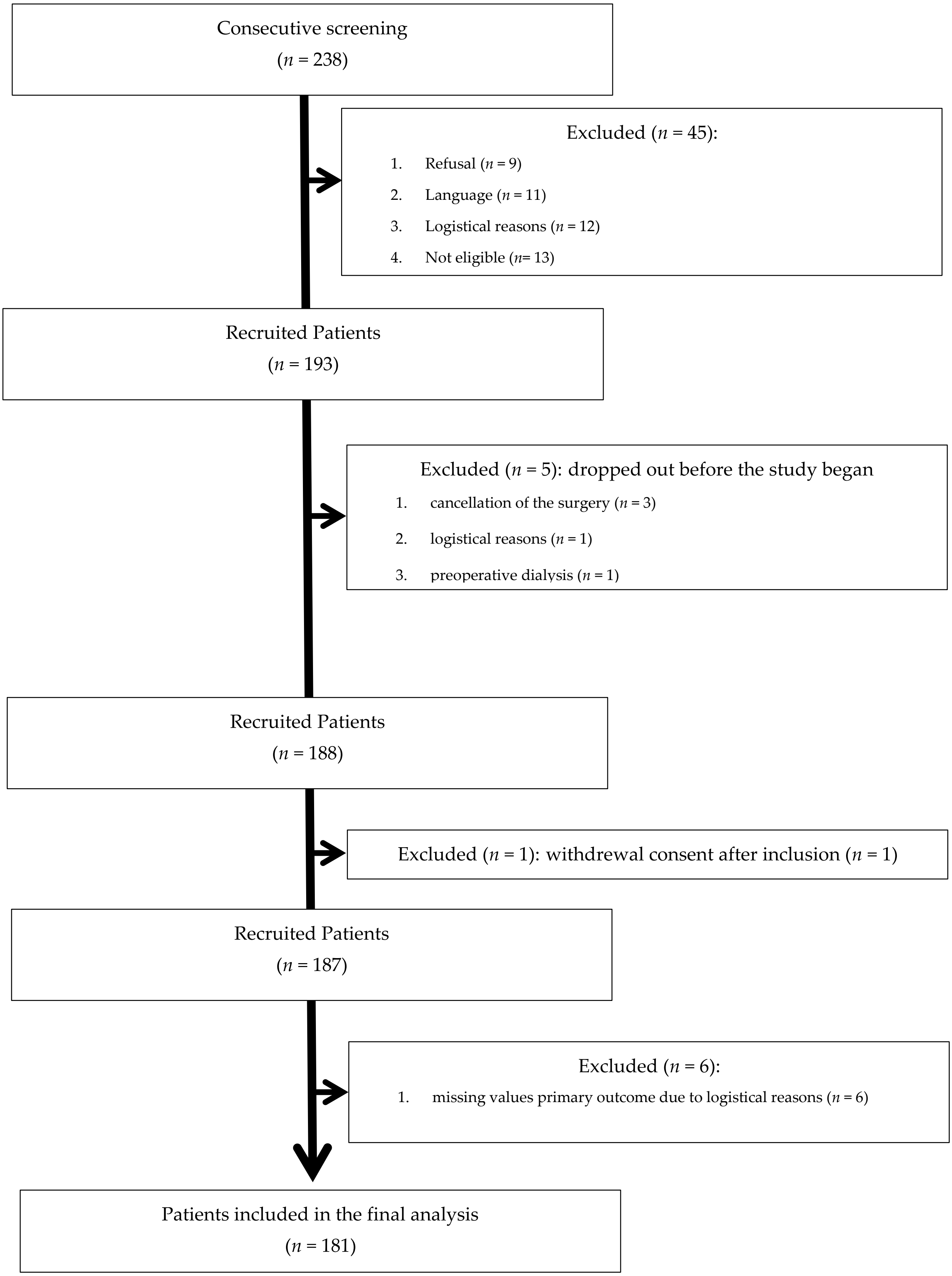

Supplement: Supplementary file 1 [file jcm-10-05817-s001.zip › jcm-1427451-supplementary.pdf]
